# Spectral Transmittance Analysis of Different Sunscreens Used in Daylight Photodynamic Therapy

**DOI:** 10.1111/phpp.70040

**Published:** 2025-07-29

**Authors:** Tamara Gracia‐Cazaña, Ana Sánchez‐Cano, Justiniano Aporta, Yolanda Gilaberte

**Affiliations:** ^1^ Department of Dermatology Miguel Servet University Hospital, IIS Aragón Zaragoza Spain; ^2^ Department of Medicine, Psychiatry and Dermatology University of Zaragoza Zaragoza Spain; ^3^ Department of Applied Physics University of Zaragoza Zaragoza Spain

**Keywords:** daylight, filters, photodynamic therapy, sunscreen

## Abstract

**Introduction:**

The protocol for daylight photodynamic therapy (DL‐PDT) includes the application of sunscreen with an SPF > 30 to block UV radiation and prevent sunburn during the 2 h of exposure to sunlight. Inorganic filters such as titanium dioxide and zinc oxide are not recommended, as they block the visible light needed to activate the photosensitizer. However, some sunscreens containing only organic filters can block 60% of the absorption spectrum of Protoporphyrin IX (PpIX).

**Materials and Methods:**

Different sunscreens commonly used to perform DL‐PDT in clinical practice were investigated by measuring their spectral transmittance. Skydome irradiances were used to calculate the absorption of PpIX, combined with these sunscreens. To perform a comprehensive analysis of this activation cream in combination with them, five distinct spectral regions were delineated for examination based on specific regions of interest (ROI): R‐I (*λ* < 475 nm), R‐II (475 ≤ *λ* < 525 nm), R‐III (525 ≤ *λ* < 560 nm), R‐IV (560 ≤ *λ* < 610 nm), and R‐V (610 nm ≤ *λ*).

**Results:**

The results indicate significant variations in the spectral transmittance and PpIX activation efficiency across different sunscreens. In the R‐I region, which is critical for PpIX activation, the PpIX activation without sunscreen was highest at 85%, while the addition of sunscreens drastically reduced activation, ranging from 28% to 8%, depending on the sunscreen. In regions from R‐II to R‐V, the activation levels were consistently low across all sunscreens evaluated. They showed similar values of approximately 5% in R‐II, 3% in R‐III, 2% in R‐IV, and 1% in R‐V, slightly lower than the corresponding PpIX alone values of 7%, 4%, 3%, and 1%, respectively.

**Conclusion:**

There are significant differences between sunscreens in terms of their absorption in the PpIX absorption spectrum. These differences do not depend on whether or not they contain inorganic filters but depend on the absorption spectrum of the filters contained in their formulation. These results highlight the impact of sunscreen selection on the efficacy of PpIX activation in DL‐PDT.

## 1 Introduction

Daylight photodynamic therapy (DL‐PDT), which emerged in Europe around 2006 as a treatment for actinic keratosis (AK), involves the application of aminolevulinic acid (ALA) or its derivative methyl‐aminolevulinate (MAL), which are precursors to the photosensitizer protoporphyrin IX (PpIX). Upon exposure to a targeted light source such as sunlight, PpIX is activated to induce selective cytotoxic effects in dysplastic or neoplastic cells. PS, visible light, and oxygen are the three key elements in DL‐PDT, and the combination of the three results in tumor necrosis and apoptosis.

Its protocol includes the application of a chemical sunscreen with an SPF > 30 to block UV, hence preventing sunburn during the 2 h daylight exposure [1]. The recommendation for using SPF30 in daylight DL‐PDT primarily aims to provide sufficient UV protection to prevent sunburn during the required daylight exposure while minimizing blockage of the spectral region necessary for PS activation. The SPF30 threshold aligns with guidelines to ensure an effective balance between protection and light transmittance. In order not to block the visible light needed to activate a particular PS, the Protoporphyrin IX (PpIX), a chemical sunscreen is recommended [1], and inorganic filters such as titanium dioxide and zinc oxide are not recommended. However, there are sunscreens containing only chemical filters that can block 60% of the PpIX absorption spectrum [2]; even there are new sunscreens in the market that contain filters that absorb in the long UVA range [3] and in the high energy visible (HEV) light range [4].

Based on the SPF classification, it has been described that SPF50+ sunscreens offer superior erythema protection compared to lower SPF products, maintaining high efficacy even in intense UV conditions and outperforming SPF15 in preventing redness [5, 6]. Although SPF testing, especially for SPF50+ products, shows considerable variability across different laboratories, with reported values ranging from 5.5 to 62.4, no significant differences in erythema prevention were observed when these sunscreens were exposed to natural sunlight compared to other high‐SPF products [7]. In this sense, SPF50 sunscreens are widely used for their higher UV protection, and studying their possible impact on PpIX absorption could help provide evidence‐based recommendations for balancing photoprotection with effective light transmittance in DL‐PDT.

In this study, our objective was to examine whether sunscreens containing newer filters that differently absorb in the long UVA and HEV light ranges block the PpIX absorption spectrum more significantly than the sunscreens currently used in DL‐PDT. As PpIX activation relies on the transmission of specific wavelengths of visible light, any formulation that overly attenuates this light could hinder the treatment's effectiveness. Therefore, if these newer sunscreens are found to block a substantial portion of the PpIX absorption spectrum, they may need to be avoided in clinical practice for DL‐PDT to ensure optimal therapeutic outcomes. Our study aims to provide clarity on this issue by evaluating from spectral data the impact of these sunscreens on PpIX absorption considering measured daylight spectra, thereby offering evidence‐based recommendations for sunscreen selection during DL‐PDT treatments.

## 2 Material and Methods

### 2.1 Spectral Skydome Measurements

**FIGURE 1:**
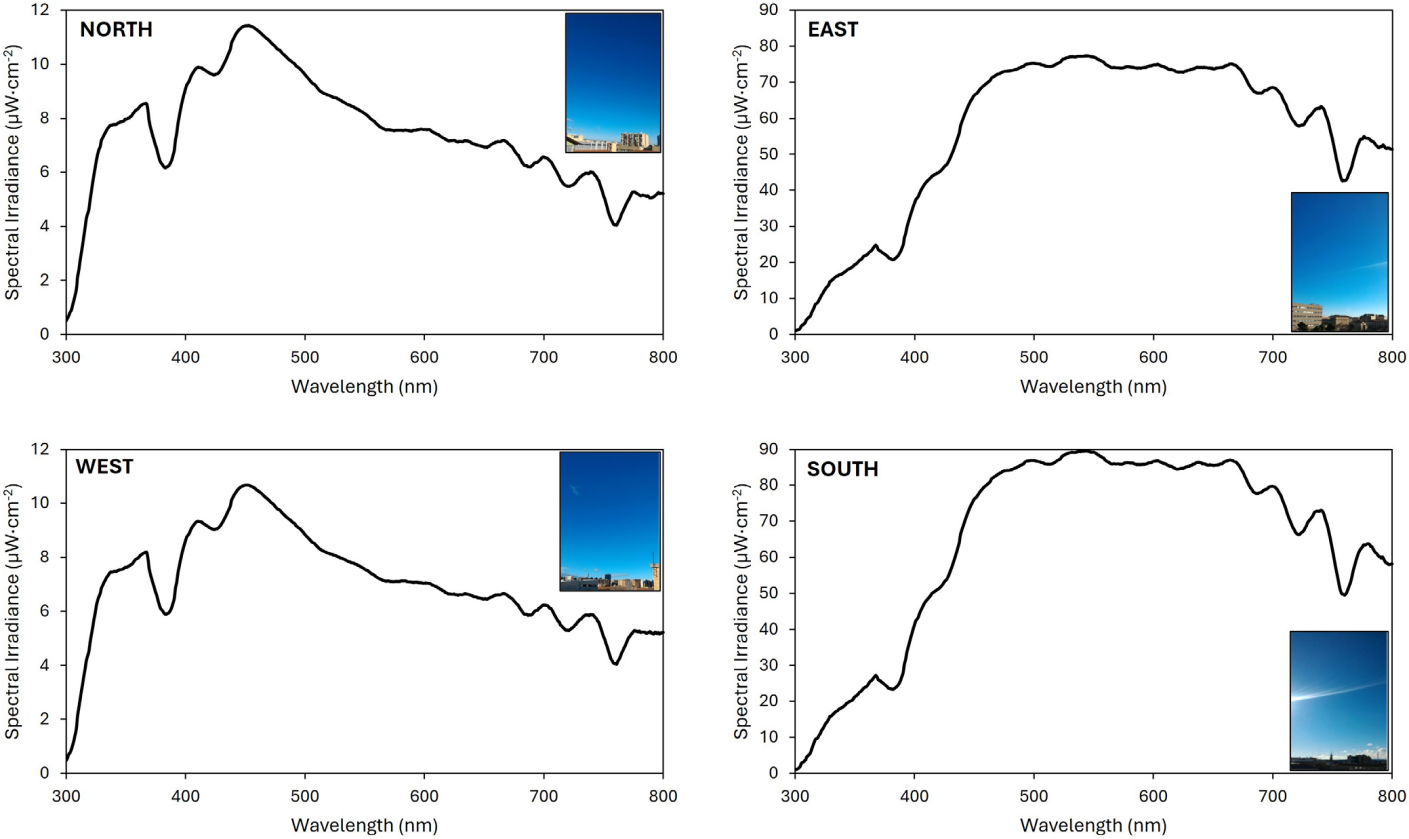
Absolute spectral irradiance (μW cm^−2^) measurements at four cardinal directions (North, East, West, South) in Zaragoza (at 30° elevation), Spain, in January. Images show representative sky images at the time of measurement.

Over the course of 1 year, multiple spectral irradiance measurements of the skydome on approximately the 15th day of each month were performed, focusing on sunny conditions and conducting measurements between 10:00 and 11:00 a.m. The experiment was located at the roof of the Sciences Faculty at the University of Zaragoza in Spain (41°3803100 N, 0°5306000 W; 243 m above sea level). The calibrated spectroradiometer employed for experimental measurements was the StellarNet‐Black Comet model (StellarNet Inc., Tampa, Florida, USA) with C20080502 calibration and NIST traceability. This instrument was utilized to assess spectral power distribution (SPD) in irradiance across the 350–800 nm range, with 1 nm increments for analysis. To ensure precision, a tripod was employed to facilitate spectrum measurements at the horizontal plane, as well as at 15°, 30°, and 45° angles in the four cardinal directions (north, south, east, and west). To document weather‐related data, photographs of the sky were captured, as shown in Figure 1 corresponding to January, and elevation 30°. Throughout the study period, sky conditions predominantly remained clear, with occasional instances of intermediate skies displaying visible sun and a limited presence of clouds, as described by Ezpeleta et al. [8]. The data obtained and detailed in this paper were used to calculate the results presented in this study.

### 2.2 Spectral Sunscreen Measurements

**FIGURE 2:**
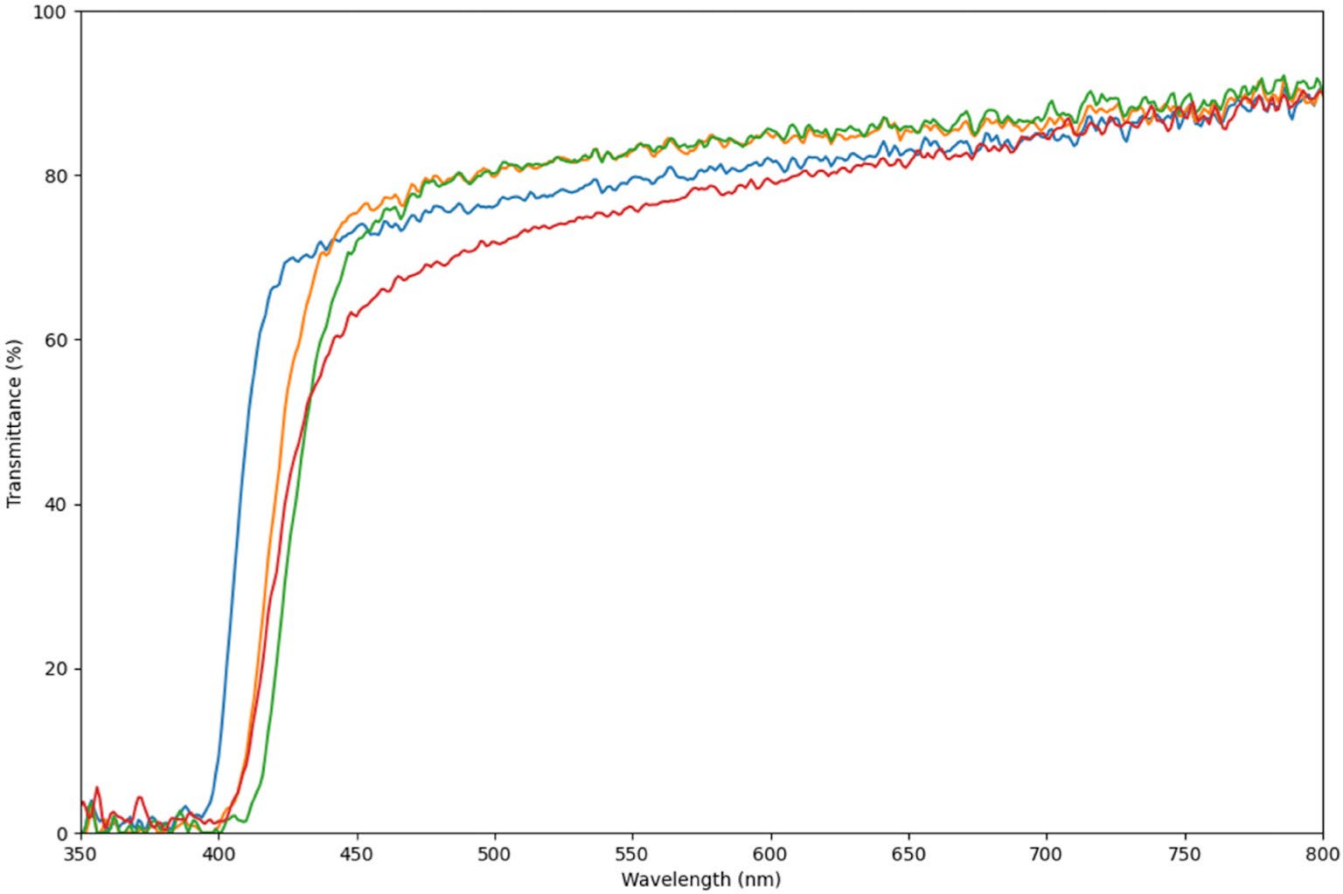
Transmittance spectra (%) of the evaluated sunscreens. Blue: CETAPHIL‐PRO OIL CONTROL MOISTURIZING SPF30, orange: ANTHELIOS UVMune 400 SPF50+ (LAROCHE), red: ACTINICA LOTION SPF 50+, and green: PROTECT EXTREME (AVENE). Values normalized to a transmittance of 90% at 800 nm for comparison.

Spectral transmittance τ (*λ*) of four selected sunscreens, namely CETAPHIL‐PRO OIL CONTROL MOISTURIZING SPF30 (Galderma, Paris, Spain), ANTHELIOS UVMune 400 SPF50+ (La Roche‐Posay, France), ACTINICA LOTION SPF 50+ (Galderma, Paris, Spain), and PROTECT EXTREME (Avène, Paris, Spain), was individually assessed for each wavelength using the previously mentioned spectroradiometer. The instrument was utilized to quantify the direct spectral transmittance of the sunscreens across the range of 350–800 nm and, subsequently, these values were normalized to a transmittance of τ (*λ* = 800 nm) = 0.90. Normalization of transmittance values at 800 nm was essential to account for variability in sunscreen application, such as cream density, inconsistent distribution, and uneven spreading, ensuring reliable comparison of spectral transmittance properties independent of application‐related variabilities. Additionally, the spectra were rescaled to enable effective inter‐comparison, further mitigating the impact of non‐uniform sunscreen distribution on the microscope slides used during the measurements and ensuring that the results accurately reflect the true performance of the sunscreens (Figure 2).

### 2.3 Theoretical Considerations

The PpIX exhibits distinctive spectral properties that are crucial to its biological and clinical significance. This porphyrin derivative demonstrates strong absorption characteristics within the visible light spectrum, from 300 to 800 nm, with prominent peaks typically observed around 400–420 nm (Soret band) and 480–630 nm (4Q bands). PpIX's absorption bands, assigned to the Soret and Q‐bands, arise from electronic transitions within its conjugated ring system, enabling efficient light energy capture and establishing its significance in diverse biological processes, particularly photodynamic therapy. Additionally, its unique fluorescence behavior is noteworthy, as PpIX can emit fluorescence upon absorption of specific wavelengths of light, particularly in the red region around 630–700 nm. Understanding these spectral attributes of PpIX is integral to its roles in cellular processes and its applications in dermatological contexts. To conduct a comprehensive analysis of this activation cream, five distinct spectral regions were delineated for examination based on specific regions of interest (ROI): R‐I (*λ* < 475 nm), R‐II (475 ≤ *λ* < 525 nm), R‐III (525 ≤ *λ* < 560 nm), R‐IV (560 ≤ *λ* < 610 nm), and R‐V (610 nm ≤ *λ*).

A custom‐written program, using the annual SPDs previously measured, was used to convert the skydome irradiance values to PpIX‐weighted irradiance E_PpIX_(μW cm‐2) obtained by multiplying the experimental averaged SPD annual skydome data and PpIX absorption function from data previously measured [9] (Equation 1). Then, the percentage of total irradiance belonging to each ROI was calculated.

(1) $$E_{\text{PpIX}}\left(\frac{\mathrm{\mu W}}{{\mathrm{cm}}^{2}}\right)=\int_{\mathrm{ROI}}\mathrm{SPD}\left(\lambda \right)\cdot \text{PpIX}\left(\lambda \right)\cdot d\lambda$$

To investigate the potential interactions between PpIX and sunscreens during PT under daylight conditions, the same averaged SPD irradiances, and the absorption spectrum of PpIX, combined with the transmittance spectrum τ (*λ*) of each analyzed sunscreen, were also calculated as follows (Equation 2).

(2) $$E_{\text{PpIX}+\text{sunscreen}}\left(\frac{\mathrm{\mu W}}{{\mathrm{cm}}^{2}}\right)=\int_{\mathrm{ROI}}\mathrm{SPD}\left(\lambda \right)\cdot \text{PpIX}\left(\lambda \right)\cdot \tau \left(\lambda \right)\cdot d\lambda$$

## 3 Results

**FIGURE 3:**
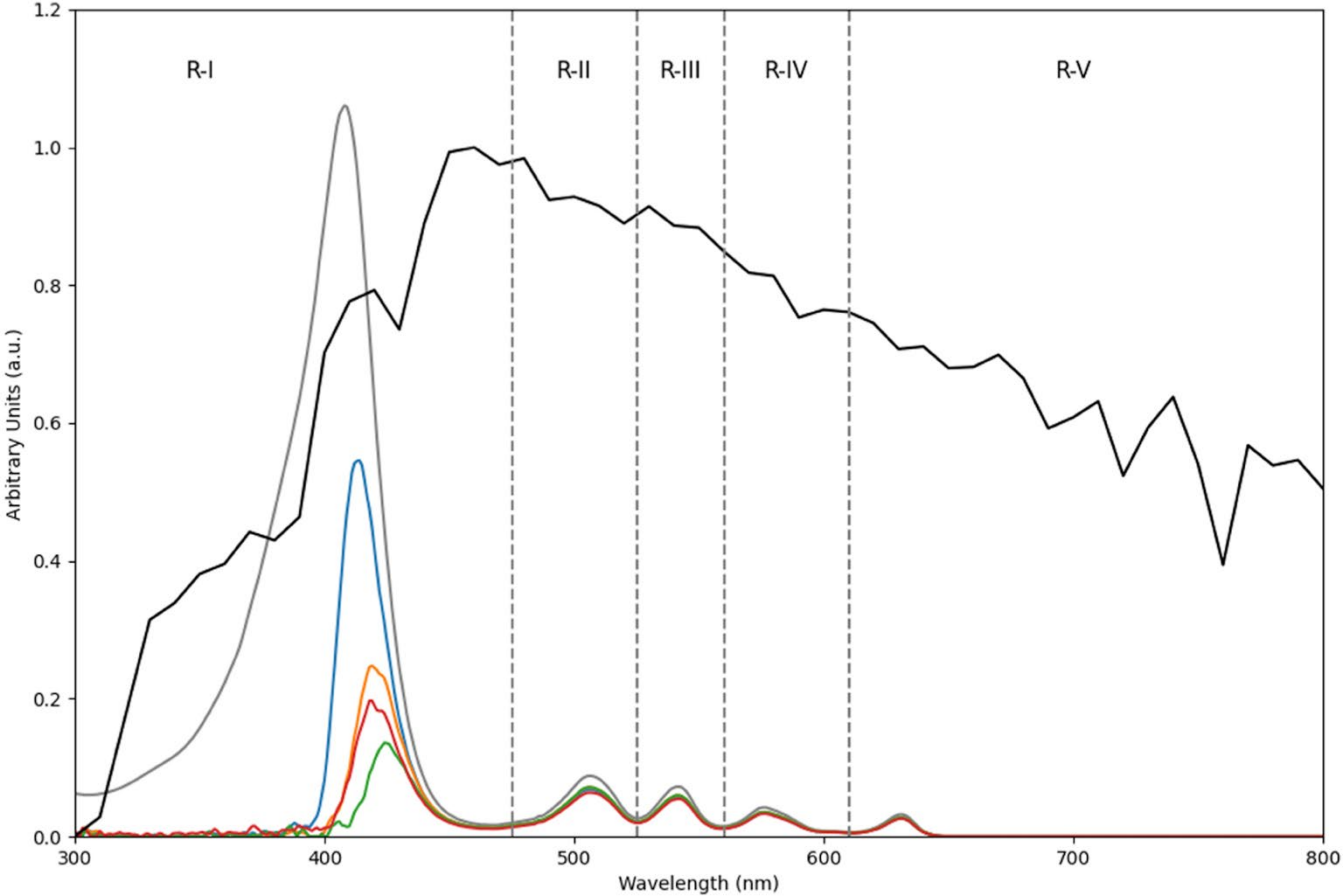
Absorption spectra of the PpIX and the combination of PpIX and the evaluated sunscreens. Grey: PpIX, blue: PpIX+CETAPHIL‐PRO OIL CONTROL MOISTURIZING SPF30, orange: PpIX + ANTHELIOS UVMune 400 SPF50 + (LAROCHE), red: PpIX+ACTINICA LOTION SPF 50+, and green: + PROTECT EXTREME (AVENE). Additionally, normalized spectral power distribution from D65 standard illuminant, representative of specific skydome conditions, is displayed in gray for comparative analysis. Vertical lines indicate spectral regions R‐I (*λ* < 475 nm), R‐II (475 ≤ *λ* < 525 nm), R‐III (525 ≤ *λ* < 560 nm), R‐IV (560 ≤ *λ* < 610 nm), and R‐V (610 nm ≤ *λ*).

Absorption spectrum of PpIX and PpIX in conjunction with each of the analyzed sunscreens is presented in Table 1 showing the percentage of the absorption spectrum distributed in every ROI previously described. The results indicate significant variations in the spectral transmittance and PpIX activation efficiency across different sunscreens. In the R‐I region (*λ* < 475 nm), which is critical for PpIX activation, the activation without sunscreen, PpIX alone, was highest at 85%, while the addition of sunscreens drastically reduced activation, with CETAPHIL achieving 28%, and UVMune, Actinica, and Protect Extreme showing further decreases to 13%, 12%, and 8%, respectively. In the R‐II region (475 ≤ *λ* < 525 nm), all sunscreen samples showed comparable activation levels of around 5%, significantly lower than the PpIX alone at 7%. Regions R‐III to R‐V (525 ≤ *λ*) exhibited minimal differences, with activation levels between 1% and 4% across all conditions. Overall, considering that the PpIX alone retained 100% activation, the addition of sunscreens reduced activation progressively, with total activation ranging from 40% in Cetaphil to 20% in Protect Extreme. Additionally, the spectrum of daylight illumination, standard D65 illuminant, is incorporated in Figure 3 to enable a graphical comparative analysis between incident light and the absorption characteristics of the various combinations.

**TABLE 1:** Distribution of the action spectra belonging to the PpIX and the PpIX in combination with the evaluated sunscreens.

	PpIX	PpIX and			
		Sample1	Sample2	Sample3	Sample4
R‐I (*λ* < 475 nm)	85%	28%	13%	12%	8%
R‐II (475 ≤ *λ* < 525 nm)	7%	5%	5%	5%	5%
R‐III (525 ≤ *λ* < 560 nm)	4%	3%	3%	3%	3%
R‐IV (560 ≤ *λ* < 610 nm)	3%	2%	2%	2%	2%
R‐V (610 nm ≤ *λ*)	1%	1%	1%	1%	1%
	100%	40%	25%	23%	20%

*Note:* Sample1: CETAPHIL‐PRO OIL CONTROL MOISTURIZING SPF30; Sample2: ANTHELIOS UVMune 400 SPF50+ (LAROCHE); Sample3: ACTINICA LOTION SPF 50+; and Sample4: PROTECT EXTREME (AVENE).

In the scenario of January data as an example, with an orientation toward the East at an angle of 30° with respect to the horizontal plane, an illuminance level of 54,631 lx was recorded. Under direct sunlight conditions, the skydome irradiance measured at 27169.1 μW cm^−2^. Results are shown in Table 2, and upon the application of PpIX, there is an overall absorption of approximately 8% in comparison to the incident irradiance without the cream, resulting in a measured total value of 2147.8 μW cm^−2^. This absorption is distributed across the previously defined spectral regions as follows: 1724.0 μW cm^−2^ (80%) in region I (R‐I), 188.8 μW cm^−2^ (9%) in region II (R‐II), 114.6 μW cm^−2^ (5%) in region III (R‐III), 80.2 μW cm^−2^ (4%) in region IV (R‐IV), and 40.2 μW cm^−2^ (2%) in region V (R‐V). Upon further introduction of the sunscreens, modifications in the values occur as presented in Table 2. By examining PpIX absorption over the evaluation period of a year with the described conditions of orientation and angles, an annual average of 10% of daylight irradiance can be utilized for straightforward computation of the cream's impact.

**TABLE 2:** Irradiance reaching at a 30° plane from the horizontal by Skydome, PpIX, and PpIX in combination with the evaluated sunscreens.

Irradiance (μW·cm^−2^)	Skydome	PpIX	PpIX and			
			Sample1	Sample2	Sample3	Sample4
R‐I (*λ* < 475 nm)	5933.0	1724.0	592.4	304.7	256.0	183.5
R‐II (475 ≤ *λ* < 525 nm)	3723.0	188.8	145.0	152.1	136.0	152.3
R‐III (525 ≤ *λ*< 560 nm)	2686.4	114.6	90.2	94.4	86.3	94.5
R‐IV (560 ≤ *λ*< 610 nm)	3713.0	80.2	64.6	67.3	62.7	67.5
R‐V (610 nm ≤ *λ*)	12159.8	40.2	33.0	34.1	32.5	34.3
Total	27169.1	2147.8	925.2	652.6	573.4	532.1

*Note:* Sample1: CETAPHIL‐PRO OIL CONTROL MOISTURIZING SPF30; Sample2: ANTHELIOS UVMune 400 SPF50+; Sample3: ACTINICA LOTION SPF 50+; and Sample4: PROTECT EXTREME.

## 4 Discussion

The introduction of DL‐PDT has been an important novelty in dermatological therapeutics, because it maintains the standard efficacy of conventional PDT in actinic keratosis (AK) while simplifying the classical technique and substantially improving its tolerability [10]. The only currently available treatment protocol has been proposed by Wiegell et al. [11] and the use of photoprotection is recommended in this protocol. The most important purpose of this recommendation is to avoid the harmful effects of UV radiation during exposure to sunlight. Obtained from other studies of DL‐PDT, where photoprotectors with different SPF (15, 20, 30, and 50) were used, similar rates of lesion resolution were observed [12, 13, 14, 15].

By the other hand, according to the protocol, it is essential that the sunscreen does not contain inorganic filters, which in addition to blocking UV radiation, can block part of the spectrum in the long UVA range and in the visible light necessary for the PpIX activation. However, although there is a list of accurate sunscreens for DL‐PDT provided by the manufacturer of the PS, we can see in this study that the choice of one or another may compromise part of the absorption range of the PpIX which in the end might influence the effectiveness of the technique.

Based on these facts, a sunscreen with SPF > 30 should be used during DL‐PDT to block UV radiation and prevent sunburn over the 2‐h exposure period. To ensure only UV is blocked while allowing visible light necessary for PpIX activation, a chemical (organic) sunscreen should be used. Sunscreens containing physical (inorganic) filters, such as zinc oxide, titanium dioxide, or iron oxide, should be avoided as they can reflect visible light, potentially reducing PpIX activation by daylight and thereby decreasing DL‐PDT efficacy. Therefore, any SPF 30+ sunscreen with organic filters is suitable for DL‐PDT. By contrast, the sunscreen Actinica (SPF 50+), containing only organic filters, was used in clinical trials and is recommended by consensus guidelines [16, 17]. In order to advance in this issue, this study aims to explore whether the filter type affects DL‐PDT efficacy, focusing specifically on the absorption spectrum of the filters within the sunscreen, rather than simply whether they are organic or inorganic, and the SPF level of protection.

In biological tissues like the skin, the depth of penetration is significantly influenced by the wavelength of the light. Shorter wavelengths, such as UV and visible light, are absorbed primarily by the outer layers of skin, leading to more superficial interaction. Administration of PpIX proves advantageous due to its absorption profile, notably within the defined R‐I (*λ* < 475 nm) region in this study. However, when considering combinations with sunscreens, caution is necessary as these screens diminish these absorption properties. In this case, our measurements emphasize the critical role of sunscreen selection when calculating minimum required doses, given the time‐dependent nature of this parameter.

According to our results, Cetaphil PRO‐OIL SPF30 was the sunscreen that competes with the PpIX absorption spectrum, followed by UVmune 400, Actinica lotion, and finally Extrem Protect. It is surprising that UVmune 400 behaves better in terms of absorbing less of the PpIX absorption spectrum than Actinica lotion, one of the sunscreens most used to perform DL‐PDT because it does not contain physical filters that absorb also in the UVA range (bis‐ethylhexyloxyphenol methoxyphenyl triazine [peaks of absorption 305, 340 nm], ethylhexyl triazone, isoamyl p‐methoxycinnamate, ethylhexyl methoxycinnamate, methylene bis‐benzotriazolyl tetramethylbutylphenol, and butyl methoxydibenzoylmethane). It is not clear what constitutes a good percentage of PpIX absorption blocking, and our findings suggest that many sunscreens reduce PpIX activation by more than 50% in vitro. For example, Cetaphil PRO‐OIL SPF30, which allowed approximately 40% residual activation, may preserve partial DL‐PDT efficacy, whereas Extrem Protect, with only ~20% residual activation, showed minimal activation. These results indicate a potential correlation between the degree of spectral filtering and the retention of DL‐PDT activity, although the clinical significance of this relationship requires further investigation. Our analysis reveals that Extrem Protect should be avoided during the DL‐PDT but could be the most appropriate to see 48 h after the treatment in terms of preventing the activation of the PpIX that will be synthesized in the area of treatment.

In contrast, longer wavelengths exhibit improved penetration capabilities, encountering reduced scattering and absorption by cellular components within the skin. This property renders longer wavelengths particularly valuable for non‐invasive treatments targeting deeper tissue layers; the higher numbered spectral regions of our study could be beneficial for these purposes. In addition, sunscreen usage does not alter the absorption of longer wavelengths, and its impact on PpIX behavior is minimal. Thus, the presented data can be useful for approximating doses required for non‐superficial applications. For a comprehensive analysis, and in scenarios where penetrating deeper skin layers holds significance, the percentages provided in Table 1 offer a valuable means of estimating both specific exposure times and doses.

O'Mahoney et al. [2] tested the effects of similar sunscreens, not the same, on DL‐PDT efficacy, focusing on different light wavebands. Our study highlights that SPF50+ sunscreens with newer filters may interfere with PpIX activation by attenuating HEV light, especially recommending the usage of products like Cetaphil PRO‐OIL SPF30, which significantly conserve the PpIX absorption spectrum. In contrast, the study by O'Mahoney et al. [2] evaluates sunscreens and window glass, finding that even organic sunscreens can block critical wavelengths for PpIX, with some sunscreens reducing PpIX‐effective doses by 38%–92%. It seems important to highlight that both studies emphasize careful sunscreen selection, as inappropriate choices can markedly impact DL‐PDT's therapeutic light dose and overall treatment efficacy.

Our study represents a significant advancement in the field by not only expanding understanding of sunscreen compatibility with PDT but also by employing an innovative methodological approach. By using a spectroradiometer to measure the spectral transmittance of each sunscreen across different wavelengths, we provide a precise, data‐driven analysis of how different sunscreens affect the availability of light required for PpIX activation. Dividing the PpIX absorption spectrum into five specific ROIs and examining each sunscreen's impact on each region enables a nuanced assessment of their potential to interfere with PDT efficacy. Importantly, we identified that certain sunscreens, particularly Cetaphil PRO‐OIL SPF30, overlap its transmittance significantly with the PpIX absorption spectrum, potentially conserving PDT effectiveness, followed by UVmune 400, Actinica lotion, and Extrem Protect. This insight equips clinicians with evidence to recommend sunscreens that minimize interference with PDT. Ultimately, our study underscores the critical role of sunscreen selection in optimizing PDT outcomes, especially by avoiding formulations with HEV light absorption, ensuring that patients receive the most effective possible treatment. The UV‐filtering properties of sunscreens, particularly in the UVA1 (340–400 nm) and UVB (280–320 nm) ranges, reduce the activation of PpIX during solar exposure. The extent to which different sunscreens attenuate these wavelengths can influence the effectiveness of DL‐PDT. Further research examining the activation of PpIX across its full absorption spectrum and correlating these findings with in vivo outcomes would enhance the selection of sunscreens for optimized DL‐PDT.

## 5 Conclusions

There are significant differences between sunscreens in terms of their absorption in the PpIX absorption spectrum. These differences do not depend on the fact that they contain or do not contain inorganic filters, but they depend on the absorption spectrum of the filters contained in their formula. Those sunscreens that include filters that absorb HEV light should be avoided during DL‐PDT, although they could be especially recommended on the days after the treatment. The influence of blocking more or less proportion of the PpIX absorption spectrum by the sunscreens on the effectiveness of DL‐PDT by the different sunscreens is not known with precision yet.

## Author Contributions

T.G.C., A.S.‐C., J.A., and Y.G. have contributed to the preparation of manuscript and critically modified. A.S.‐C. and J.A. contributed to the preparation of figures. All authors contributed to the article and approved the submitted version.

## Disclosure

All people designated as authors had participated in the work to take public responsibility in its contents.

## Conflicts of Interest

T.G.C. has received grants for attending congresses as well as fees for studies, lectures, presentations, courses, and consultancy from Galderma and Rilastil. Y.G. has received grants for attending congresses as well as fees for studies, lectures, presentations, courses, and consultancy from Galderma, Roche Posay, Isdin, Avene, Cantabria Labs, and Rilastil.

## Data Availability

The data that support the findings of this study are available from the corresponding author upon reasonable request.
